# Protein-Based Delivery Systems for Anticancer Metallodrugs: Structure and Biological Activity of the Oxaliplatin/β-Lactoglobulin Adduct

**DOI:** 10.3390/ph15040425

**Published:** 2022-03-30

**Authors:** Daria Maria Monti, Domenico Loreto, Ilaria Iacobucci, Giarita Ferraro, Alessandro Pratesi, Luigi D’Elia, Maria Monti, Antonello Merlino

**Affiliations:** 1 Department of Chemical Sciences, University of Naples Federico II, Complesso Universitario di Monte Sant’Angelo, Via Cintia, 21, 80126 Napoli, Italy; mdmonti@unina.it (D.M.M.); domenico.loreto@unina.it (D.L.); ilaria.iacobucci@unina.it (I.I.); giarita.ferraro@unina.it (G.F.); luigi.delia@unina.it (L.D.); montimar@unina.it (M.M.); 2 CEINGE Advanced Biotechnologies s.c.a.r.l., Via G. Salvatore 486, 80145 Napoli, Italy; 3 Department of Chemistry and Industrial Chemistry, University of Pisa, Via G. Moruzzi 13, 56124 Pisa, Italy; alessandro.pratesi@unipi.it

**Keywords:** delivery system, protein, metallodrug, anticancer, protein metalation

## Abstract

β-lactoglobulin is the major component of whey. Here, the adduct formed upon the reaction of the protein with oxaliplatin (OXA) has been prepared, structurally characterized by X-ray crystallography and electrospray ionization–mass spectrometry, and evaluated as a cytotoxic agent. The data demonstrate that OXA rapidly binds β-lactoglobulin via coordination with a Met7 side chain upon release of the oxalate ligand. The adduct is significantly more cytotoxic than the free drug and induces apoptosis in cancer cells. Overall, our results suggest that metallodrug/β-lactoglobulin adducts can be used as anticancer agents and that the protein can be used as a metallodrug delivery system.

β-lactoglobulin is a whey carrier protein of 18.4 kDa [1,2]. Due to its high water solubility, safe status, biodegradable nature, gel-forming ability, abundance, stability at an acidic pH, and stability against gastric pepsin, β-lactoglobulin can be considered a good protein for the preparation of micro- or nano-particles in the pharmaceutical and food industries [3,4,5,6,7], although it is one of the major allergens in cows’ milk. Since the protein binds anticancer Pt compounds [6,7,8,9,10,11,12], it can, in principle, be used as a nanocarrier with controlled release properties for these molecules. In this respect, it has been demonstrated that β-lactoglobulin–pectin nanoparticles are able to transfer cytotoxic Pt compounds to cancer cells [13].

In-solution data indicated that cisplatin (CDDP), the most important Pt-based anticancer agent in clinical use [14,15,16,17], binds β-lactoglobulin, affecting the secondary structure content of the protein [6,7,8,9,10,11,12]. Crystallographic data demonstrated that Pt centers coordinate the side chains of Met7, His146 and even Lys8 upon the release of metal ligands [18]. Spectroscopic and spectrometric measurements suggested that β-lactoglobulin can also bind oxaliplatin (OXA) [9,11], a Pt-based anticancer agent that is approved for the treatment of colorectal cancer [19,20,21]. Docking experiments suggested that OXA binds the protein in a cavity lined by the residues Thr18, Ser21, Glu157, Glu44, Trp19, Val43, Leu46, Ile56, Phe105, Leu122 [11]. However, further experiments are needed to validate this prediction.

Herein, we study the interaction of OXA with β-lactoglobulin by solving the X-ray structure of the adduct obtained upon reaction of the anticancer drug with the protein; analyzing the adduct formation by circular dichroism; and collecting electrospray ionization mass spectra as a function of time. Furthermore, the potential use of this adduct as an anticancer agent is evaluated. The cytotoxicity of OXA/β-lactoglobulin is compared to the free drug.

Circular dichroism data of the protein in the presence of OXA are reported in Figure S1 and Figure S2. Superimposition of the spectra indicates that the protein retains its secondary structure in the presence of the metallodrug after 24 h of incubation.

Crystals of OXA/β-lactoglobulin adduct were obtained using soaking experiments: crystals of the ligand-free protein, grown using a hanging-drop vapor-diffusion method with a reservoir solution containing 35% pentaerythritol propoxylate, 0.2 M KCl, and 0.04 M HEPES at pH 7.5 (protein concentration: 30 mg mL^−1^), were soaked for 3 days in a solution of the reservoir with OXA. The final protein-to-metal ratio was about 1:3. The X-ray diffraction data were then collected at the XRD2 beamline of the Elettra synchrotron in Trieste, Italy (see SI for further details) at a 2.01 Å resolution. Analysis of the X-ray diffraction pattern indicated that β-lactoglobulin crystals grown under this condition belong to a new crystal form. The structure of OXA/β-lactoglobulin was solved by molecular replacement, using the coordinates of molecule A from the PDB code 6ZSR [18], without ligands and water molecules, as starting model.

The overall structure of the OXA/β-lactoglobulin adduct is reported in Figure 1. The final structure contained two molecules in the asymmetric unit (molecules A and B hereafter) and was refined to an R-factor of 0.212 (R-free = 0.284) (see SI for further details).

As expected on the basis of the statistical analysis carried out comparing the structures of the Pt-protein adducts with those of metal-free proteins [22], the overall conformation of β-lactoglobulin is not significantly affected by OXA binding. The Cα root mean square deviation of chains A and B in our structures (PDB code 7NQB) from those of the ligand-free protein (PDB code 1BEB) is within the range 0.43–0.54 Å. An inspection of the difference using Fourier and anomalous electron-density maps clearly reveals the presence of a peak due to the binding of a Pt-containing fragment close to the side chain of Met7 of molecule A (Figure 2), in agreement with what is observed in the structure of the adduct formed when crystals of the protein have been treated with CDDP [18]. Access to the side chain of Met7 of molecule B is hampered by the crystal lattice. The Pt center coordinates the S atom of the side chain of Met (SD atom) with a Pt-SD distance of 2.48 Å. Ligands of Pt are not well defined.

To obtain further insights into the OXA fragment(s) that can bind the protein, electrospray ionization mass spectrometry data were collected upon 0 h, 3 h, 9 h, 18 h, 33 h and 72 h of incubation. The spectrum of the free protein (0 h) shows the presence of two species at a molecular weight of 18,276.67 ± 0.40 Da and 18,362.46 ± 0.52 Da, corresponding to the B and A β-lactoglobulin variants, respectively (Figure 3A), in agreement with previous studies [18]. Both variants bind OXA (Table S1, Supplementary Materials).

At shortest-reaction-time (3 h), ESI-MS analysis reveals that both β-lactoglobulin variants bind OXA, as suggested by the detection of species with a molecular weight of 18,674.96 ± 0.67 Da and 18,759.92 ± 0.86 Da, respectively (Figure 3B). The detected Pt-containing fragment bound to the protein is compatible with the non-covalent binding of OXA to β-lactoglobulin or with the typical activation pattern of OXA, characterized by a ring opening and the subsequent oxalate release [23,24].

After 9 h of incubation, a small amount of both isoforms binds two molecules of the drug (19,069.13 ± 1.20 Da for B variant, and 19,157.68 ± 0.89 Da for A variant). These species do not accumulate over time and, starting from 9 h of incubation, a species showing a molecular weight of 18,583.11 ± 1.64 Da appears. This signal was assigned to the adduct of the B variant bound to the [Pt(DACH)]^2+^ fragment (DACH = 1,2-diaminocyclohexane) (Figure 3C). The amount of the [Pt(DACH)]^2+^/β-lactoglobulin adduct increases over time, in terms of relative abundance with respect to the free protein, for up to 72 h (Figure 3D–F). The related species involving the A isoform was not immediately evident, since its expected molecular weight (18,672.56 Da) differs by only 2 Da from the mass of the adduct of the B variant with OXA (18,674.48 Da). However, at longest time, the decrease in the signal of the A variant bound with OXA (18,760.27 ± 0.76 Da), and the concomitant increase in the signal with a molecular weight that strictly agrees with that expected for the adduct of the A variant with [Pt(DACH)]^2+^ (18,672.47 ± 1.14 Da), confirm the formation of this species. This finding suggests the following mechanism of protein metalation [25]: the drug binds to β-lactoglobulin, coordinating protein-residue side chains upon the complete release of the oxalate moiety. This picture is in agreement with what was expected on the basis of previous literature data [23,24,25,26,27,28].

In vitro cytotoxicity of the OXA/β-lactoglobulin adduct was performed on three human cancer cell lines (A431, Caco-2 and HT29) and an immortalized human cell line (HaCaT). The adduct was obtained by incubating the protein with the metallodrug in a 1:10 protein-to-metal ratio for 24 h, then removing the excess of OXA using dialysis (see SI for further details). The ICP-AES data and the results of the BCA assay reveal that the adduct contains Pt in a 1:1 ratio with the protein.

The MTT assay was performed after a 72 h incubation, and the results are reported in Figure 4. The IC_50_ values (i.e., the concentration of a molecule able to kill 50% of cells) are reported in Table 1. Interestingly, the metallodrug/protein adduct possesses IC_50_ values much lower (from 30 to more than 60 times) than those obtained for the free drug. No toxic effect was observed for the metal-free protein, up to a protein concentration of 0.27 mg mL^−1^ (15 μM).

Then, the uptake and cell death mechanism were performed on A431 cells. The uptake was performed by incubating cells in the presence of either the OXA or OXA/β-lactoglobulin adduct at the IC_50_ value. After a 3 h incubation, the amount of metal was measured using ICP-AES, and the results are shown in Table 2. Interestingly, cells incubated with OXA/β-lactoglobulin showed about double the Pt content of cells incubated only with OXA, even though the amount of OXA needed to reach the IC_50_ value was about 60 times higher that of OXA/β-lactoglobulin.

The cell death mechanism was analyzed using Western blotting. Cells were incubated with either the OXA or OXA/β-lactoglobulin adduct at the IC_50_ value for 72 h, then the involvement of apoptosis was evaluated. As shown in Figure 5, pro-caspase-3 levels significantly decreased in the presence of each drug, while pro-caspase-9 significantly decreased only in the presence of OXA/β-lactoglobulin.

In conclusion, herein, we have studied the formation of the adduct obtained upon the reaction of OXA with β-lactoglobulin. Mass spectrometry data suggest that OXA rapidly binds the protein via the coordination of a [Pt(DACH)]^2+^ fragment to a β-lactoglobulin residue side chain. The structure of the adduct is determined at a 2.01 Å resolution. There are only a few molecular structures of adducts formed upon the reaction of OXA with proteins [24,25,26,27,28]. The crystallographic data indicate that β-lactoglobulin binds OXA without altering its overall structure, and that the Pt center binds the side chains of Met7. Since the Pt binding to the Met side chain could be reversible, our data suggest that β-lactoglobulin could be used as a Pt-based drug delivery system. For this reason, we studied the potential use of the OXA/β-lactoglobulin adducts as anticancer agents. Notably, the cytotoxicity data reveal that the adduct formed upon the reaction of the anticancer agent with the protein exerts higher cytotoxicity than free drugs by inducing the same mechanism of cell death. Altogether, these results lead the way for a rational design and the development of new biomaterials based on metallodrug/β-lactoglobulin adducts or metallodrug/β-lactoglobulin nanoparticles that can potentially be administered as oral drugs.

## Figures and Tables

**Figure 1:**
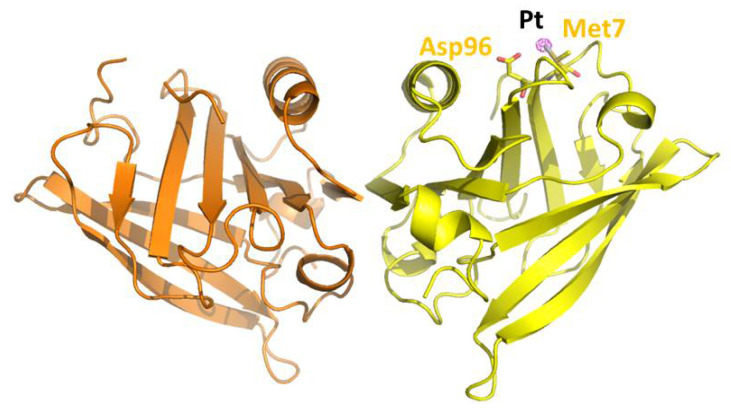
Overall structure of OXA/β-lactoglobulin adduct (PDB code 7NQB). The structure was obtained using X-ray diffraction data derived from crystals of β-lactoglobulin soaked for 3 days in a solution containing OXA. The Pt binding site was identified by inspecting an anomalous difference electron density map, reported in violet at a 10.0 σ level.

**Figure 2:**
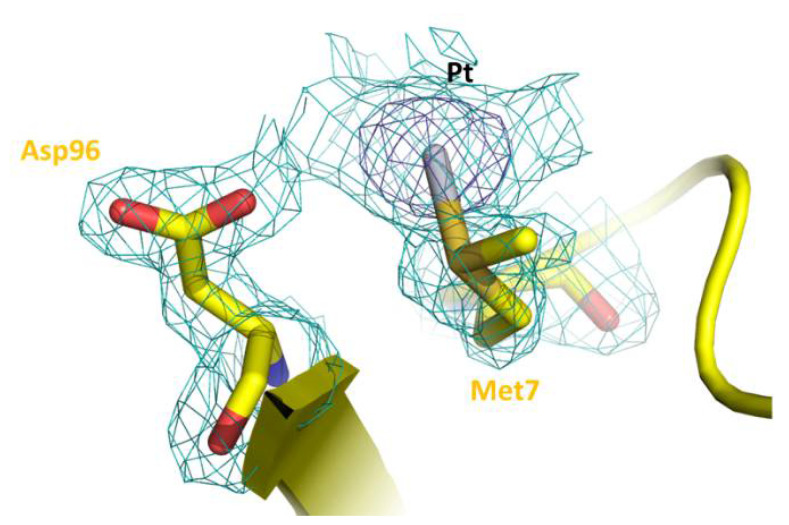
Pt-containing fragment binding site in the structure of the OXA/β-lactoglobulin adduct. Pt binds the side chain of Met7. 2Fo-Fc electron density map is contoured at 1.0 σ (cyan) and 5.0 σ (blue) level.

**Figure 3:**
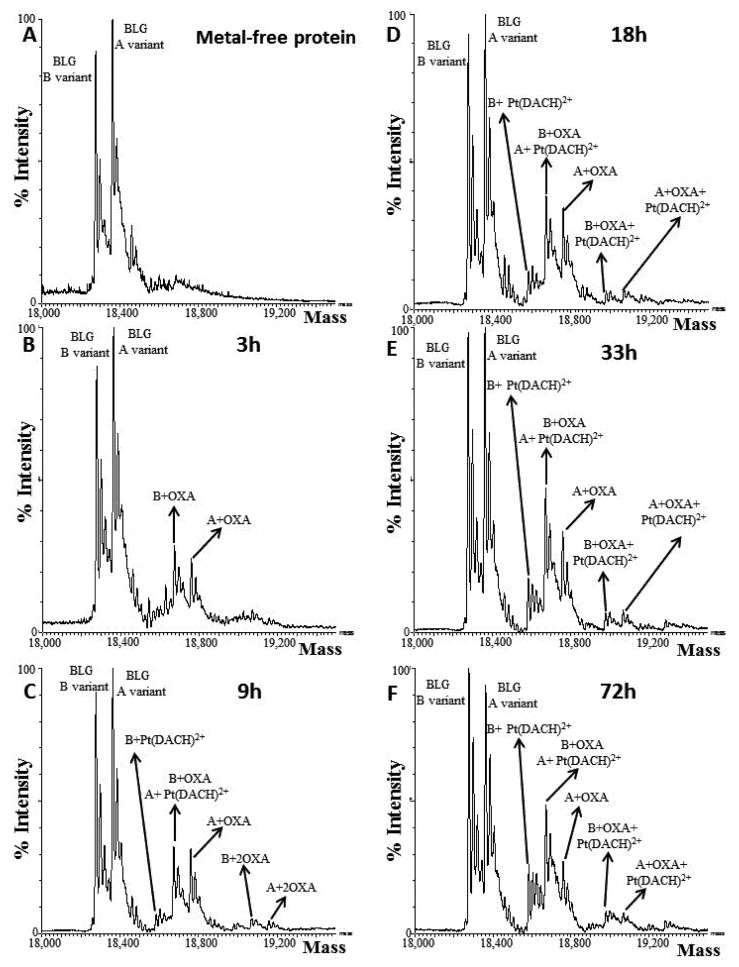
Deconvoluted ESI-MS spectra of free β-lactoglobulin (t = 0, panel **A**) and following 3 h (**B**), 9 h (**C**), 18 h (**D**), 33 h (**E**) and 72 h (**F**) of incubation with OXA. A = A variant, B = B variant.

**Figure 4:**
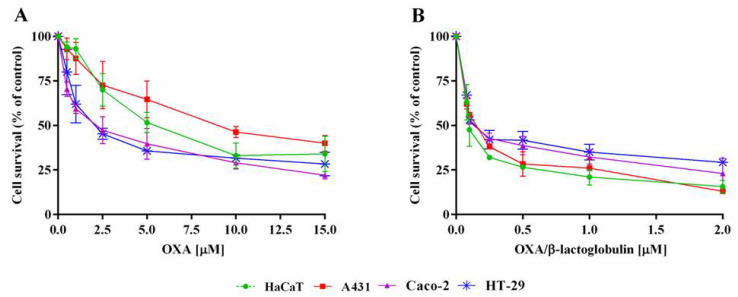
Effect of OXA/β-lactoglobulin and OXA on the survival of eukaryotic cells. Immortalized human keratinocytes (HaCaT, green circle), human epidermoid carcinoma (A431, red squares), human colorectal adenocarcinoma (Caco-2, purple triangles and HT-29, blue asterisks) were incubated with increasing amounts of OXA (**A**) or OXA/β-lactoglobulin (**B**) for 72 h. Cell viability was assessed using a 3-(4,5-dimethylthiazol-2-yl)-2,5-diphenyltetrazolium bromide (MTT) assay and expressed as described in the Materials and Methods section. Values are given as means ± SD (*n* > 3).

**Figure 5:**
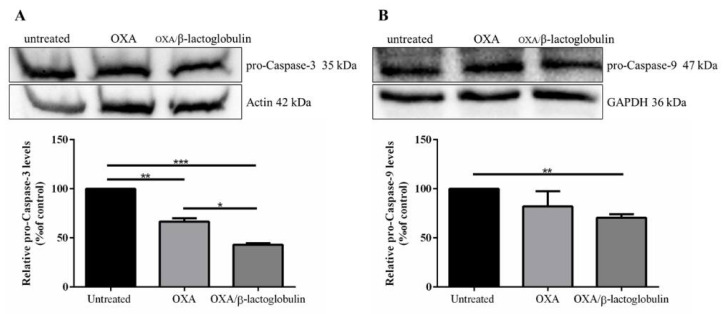
Apoptosis activation in A431 cells incubated with OXA or OXA/β-lactoglobulin. Cells were incubated in the presence of each molecule at the concentration needed to reach the IC_50_ value. After a 72 h incubation, cell extracts were prepared and subjected to Western blotting analysis using anti-pro-caspase-3 antibody (**A**), or anti-pro-caspase-9 antibody (**B**). Actin or GAPDH were used as internal standards. The relative densitometric analysis is reported below. Values are given as means ± SD (*n* > 3). * indicates *p* < 0.05, ** indicates *p* < 0.01, *** indicates *p* < 0.005.

**Table 1:** IC_50_ values for OXA, β-lactoglobulin, and the adduct OXA/β-lactoglobulin. Values are reported as metal concentration (μM). Values are given as means ± SD (*n* > 3). CDDP is reported as reference.

	A431	HT-29	Caco-2	HaCaT
OXA	8.8 ± 1.1	2.1 ± 0.6	3.4 ± 1.1	7.3 ± 2.3
OXA/β-lactoglobulin	0.149 ± 0.001	0.066 ± 0.005	0.066 ± 0.001	0.110± 0.014
β-lactoglobulin	>15	>15	>15	>15
CDDP	7.3 ± 0.1	5.9 ± 1.6	9.2 ± 1.3	3.4 ± 1.0

**Table 2:** Platinum content in A431 cancer cells measured with ICP-AES. Values are given as means ± SD (*n* = 2). Control cells are untreated cells. N.D., not detected.

Compound	Pt content (10^−9^ µg/cell)
Control	N.D.
OXA	4.00 ± 0.06
OXA/β-lactoglobulin	7.81 ± 0.08

## Data Availability

Data is available within article and Supplementary Materials.

## Supplementary Materials

The following are available online at https://www.mdpi.com/article/10.3390/ph15040425/s1. Table S1. Results of ESI-MS experiments; Table S2. Data collection and refinement statistics.; Figure S1. Circular dichroism spectra of OXA/β-lactoglobulin adduct and of the metal-free protein in 5mM Hepes pH 7.5; Figure S2. Circular dichroism spectra of OXA/β-lactoglobulin adduct (black) and of metal-free protein (red) in 10 mM ammonium acetate at pH 6.8; Figure S3. Uncropped image of western blot experiments.
