# Supplementary material for: Protein-Based Delivery Systems for Anticancer Metallodrugs: Structure and Biological Activity of the Oxaliplatin/β-Lactoglobulin Adduct
Source: Pharmaceuticals (Basel). 2022 Mar 30;15(4):425. doi: 10.3390/ph15040425 (PMC9033069; doi:10.3390/ph15040425)
Supplement: Supplementary file 1 [file pharmaceuticals-15-00425-s001.zip › pharmaceuticals-1624465-supplementary.pdf]

Supporting information

Protein-based delivery systems for anticancer metallodrugs: structure and biological activity of the oxaliplatin/β-lactoglobulin adduct

Daria Maria Monti,[1] Domenico Loreto, [1] Ilaria Iacobucci, [1,2] Giarita Ferraro,[3] Alessandro Pratesi,[4] Luigi D'Elia,[1] Maria Monti, [1,2] and Antonello Merlino [1*]

1 Department of Chemical Sciences, University of Naples Federico II, Complesso Universitario di Monte Sant'Angelo, Via Cintia, 21, I-80126, Napoli, Italy
2 CEINGE Advanced Biotechnologies s.c.a r.l., Via G. Salvatore 486, 80145, Napoli, Italy
3 Department of Chemistry "Ugo Schiff", University of Florence, via della Lstruccia, 3-13, Sesto Fiorentino, Firenze, Italy
4 Department of Chemistry and Industrial Chemistry, university of Pisa, Via G. Moruzzi 13, 56124, Pisa, Italy
* Correspondence: antonello.merlino@unina.it

Table S1. Results of ESI-MS experiments. For each time of incubation, the calculated and the theoretical molecular weight of detected adducts are reported.

Table S2. Data collection and refinement statistics. Values in parentheses are for the highest resolution shell.

Figure S1. Circular dichroism spectra of OXA/β-lactoglobulin adduct and of the metal-free protein in 5mM Hepes pH 7.5.

Figure S2. Circular dichroism spectra of OXA/β-lactoglobulin adduct (black) and of metal-free protein (red) in 10 mM ammonium acetate at pH 6.8.

Figure S3. Uncropped image of western blot experiments

Materials and Methods

Bovine milk β-lactoglobulin (A+B forms) and OXA were purchased from Sigma and used without further purification. All other reagents were of analytical grade. The β-lactoglobulin concentrations in solution were determined spectrophotometrically at 280 nm using, for calculation, a molar absorption coefficient of $\varepsilon = 17{,}600\ M^{-1}\ cm^{-1}$ [1].

Crystallization, X-ray diffraction data collection, structure solution and refinement of OXA/β-lactoglobulin

Manual crystallization trials were carried out in hanging-drop at 20°C. β-lactoglobulin was crystallized by mixing 1 µl protein solution and 1 µl reservoir solution [35% pentaerythritol propoxylate, 0.2 M KCl, 0.04 M Hepes pH 7.5 ([protein]=30 mg ml^{-1})]. To obtain crystals of the adduct, crystals of β-lactoglobulin were then soaked in the crystallization solution supplemented with OXA in protein to metal ratio 1:3. Before freezing, crystals were transferred to a cryo-solution formed by the reservoir with 20 % glycerol for 1 min. Diffraction data for the adduct were collected on XRD2 beamline of Elettra, Trieste. Data were processed with Autoproc [2]. Molecular replacement was carried out using Phaser [3] and chain A from the structure of β-lactoglobulin with PDB code 6ZSR as starting model [4]. Refinement was carried out using Refmac [5] and model building using Coot [6]. Data-collection and final refinement statistics are summarized in Table S2. The structure was deposited in the Protein Data Bank under the accession code 7NQB.

Circular Dichroism analysis

Circular dichroism (CD) spectra of β-lactoglobulin and OXA/β-lactoglobulin adducts in 1:1 and 1:5 protein to metal ratio were collected with a Jasco J-715 spectropolarimeter equipped with a Peltier temperature controller. Far-UV measurements were carried out at protein concentration of 0.1 mg ml^{-1} in 5 mM Hepes pH 7.5 (Figure S1) and 10 mM ammonium acetate, pH 6.8 (Figure S2), at 10 °C, using a 0.1 cm optical path length cell. Other parameters are: 50 nm min^{-1} scanning speed, 2 s response time, 1 nm data pitch and 2.0 nm bandwidth. Spectra were obtained averaging three scans.

ESI-MS analysis

The reaction between OXA and β-lactoglobulin has been carried out in water by mixing the protein and the drug in a 3-fold OXA molar excess at 25 °C and monitored over time acquiring ESI-MS spectra upon 0 h (free protein), 3 h, 9 h, 18 h, 33 h and 72 h. Following dilution in 15 mM ammonium acetate pH 6.8, each mixture was analysed with a Q-ToF Premier (Waters, Milford, MA, USA) mass spectrometer by direct injection mode at a 10 µl min^{-1} flow rate. The source parameters were set at 3 kV for capillary voltage, 42 kV for cone voltage, and 80 °C for the temperature. The m/z acquisition range spanned from 900 to 3500 and the raw data were processed by the MassLynx 4.1 software (Waters, Milford, MA, USA), as previously reported [4].

ICP-AES analysis

ICP-AES data were collected on the OXA/β-lactoglobulin adduct, prepared incubating for 24 h at room temperature the protein in the presence of Oxaliplatin in 1:10 protein- to-metal ratio in 10 mM Hepes pH 7.5. Successively, the metalated protein was isolated by a centrifugal filter device with cut-off membrane of 10 kDa and washed several times with the same buffer. The sample was recovered in a PE vial and mineralized in a thermo- reactor at 80 °C for 8 h with 2 mL of 50% v/v diluted aqua regia (HCl suprapure grade and HNO_3 suprapure grade in a 3:1 ratio) in Milli-Q water (≥18 MΩ · cm). After that time, the sample was cooled down to room temperature and further diluted to a final volume of 6 mL with ultrapure water (≥18 MΩ · cm). The determination of platinum content in this solution was performed by a Varian 720-ES Inductively Coupled Plasma Atomic Emission Spectrometer (ICP-AES) equipped with a CETAC U5000 AT+ ultrasonic nebulizer, to increase the method sensitivity. The calibration curve of platinum was obtained using known concentrations of a Pt ICP standard solution purchased from Sigma-Aldrich. Moreover, each sample were spiked with 1 ppm of Ge used as an internal standard. The wavelength used for the Pt determination was 217.468 nm whereas for Ge the line at 209.426 nm was used. The operating conditions were optimized to obtain maximum signal intensity and, between each sample, a rinse solution of HCl suprapure grade and HNO_3 suprapure grade at a 3:1 ratio in ultrapure water was used to avoid any "memory effect". The measured Pt content was referred to the sample protein concentration determined on the

recovered protein/metal solution using the Bradford assay. For the cellular uptake experiment, A431 cells were seeded at a density of $2 \times 10^4/cm^2$ and incubated in the presence of either OXA or OXA/β-lactoglobulin adduct at the IC_{50} value for 3 h. The cellular pellets were recovered in PE vials. The determination of the platinum concentration was normalized to the cell number in each sample.

Cytotoxicity and Western blotting analyses

Human epidermoid carcinoma cells (A431), human colorectal adenocarcinoma (Caco-2, HT-29) were from ATCC, whereas human keratinocyte cells (HaCaT) were from Innoprot. Cells were cultured in Dulbecco's modified Eagle's medium (Sigma-Aldrich), with 2 mM L- glutamine, 10% fetal bovine serum, and antibiotics (HyClone, Logan, UT, United States) and grown in a 5% CO_2 humidified atmosphere at 37 °C. Cells were seeded in 96-well plates at a density of 2.5×10^3/well. 24 h after seeding, cells were incubated with increasing amount of each metallodrug/protein or metallodrug to be tested for 72 h. At the end of incubation, cell viability was assessed by the MTT assay as previously described [7]. Cell survival was expressed as percentage of viable cells in the presence of the analyzed protein adduct, with respect to control cells. Control cells are represented by cells grown in the absence of the molecule under test and by cells supplemented with identical volumes of buffer. The occurrence of apoptosis was determined by Western blot analyses, analyzing cell lysates as previously reported [7].

Statistical analysis

All measurements were performed on at least three freshly prepared samples and are reported as means and standard deviations (SD). For statistical analyses, GraphPad Prism 6.01 software for Windows (GraphPad Software Inc., San Diego, CA, USA) was used: T test was used for all the experiments.

Table S1. Results of ESI-MS experiments. For each time of incubation, the calculated and the theoretical molecular weight of detected adducts are reported.

Time	Exp. MW (Da)	Theor. MW (Da)	Species
0 h	18276.67 ± 0.40	18277.2	B variant*
	18362.46 ± 0.52	18363.3	A variant*
3 h	18674.96 ± 0.67	18674.48	B variant + OXA
	18759.92 ± 0.86	18760.58	A variant + OXA
9 h	18583.11 ± 1.64	18586.46	B variant + $Pt(DACH)^{2+}$
	18673.84 ± 1.01	18674.48	B variant + OXA/ A variant +$Pt(DACH)^{2+}$
	18760.18 ± 0.14	18760.58	A variant + OXA
	19069.13 ± 1.20	19071.76	B variant + 2 OXA
	19157.68 ± 0.89	19157.86	A variant + 2 OXA
18 h	18584.21 ± 0.29	18586.46	B variant + $Pt(DACH)^{2+}$
	18673.30 ± 0.76	18674.48	B variant + OXA
		18672.56	A variant + $Pt(DACH)^{2+}$
	18759.98 ± 0.41	18760.58	A variant + OXA
	18978.73 ± 0.86	18983.74	B variant + $Pt(DACH)^{2+}$+OXA
	19069.70 ± 0.80	19069.84	A variant + $Pt(DACH)^{2+}$+OXA
33 h	18584.21 ± 0.29	18586.46	B variant + $Pt(DACH)^{2+}$
	18673.33 ± 0.80	18674.48	B variant + OXA
		18672.56	A variant + $Pt(DACH)^{2+}$
	18759.95 ± 0.70	18760.58	A variant + OXA
	18977.96 ± 1.67	18983.74	B variant + $Pt(DACH)^{2+}$+OXA
	19069.76 ± 1.09	19069.84	A variant + $Pt(DACH)^{2+}$+OXA
72 h	18584.56 ± 0.91	18586.46	B variant + $Pt(DACH)^{2+}$
	18672.47 ± 1.14	18674.48	B variant + OXA
		18672.56	A variant + $Pt(DACH)^{2+}$
	18760.27 ± 0.76	18760.58	A variant + OXA
	18980.31 ± 1.52	18983.74	B variant + $Pt(DACH)^{2+}$+OXA
	19069.38 ± 0.96	19069.84	A variant + $Pt(DACH)^{2+}$+OXA

*These peaks are present in all ESI MS spectra that have been registered.

Table S2. Data collection and refinement statistics. Values in parentheses are for the highest resolution shell.

Data collection	
Space group	P2$_1$
a (Å)	35.87
b (Å)	112.58
c (Å)	38.08
α (°)	90
β (°)	117.64
γ (°)	90
Resolution range (Å)	56.26-2.01
Unique reflections	17918 (2486)
Completeness (%)	86.9 (92.4)
Redundancy	2.3 (1.9)
†Rmerge (%)	0.060 (0.152)
Average I/σ(I)	8.8 (3.9)
CC$_{1/2}$	0.958 (0.939)
Anom. completeness (%)	70.0 (50.0)
Wilson B-factor (Å^2)	19.3
Refinement	
Resolution range (Å)	56.29-2.01
N. of reflections (working set)	16607
N. of reflections (test set)	772
R-factor/R-free (%)	21.2/28.4
N. of atoms	2592
Average B-factors (Å^2)	
All atoms	24.96
Pt atoms	29.46 (occupancy=0.70)
R.m.s. deviations	
Bond lengths (Å)	0.007
Bond angles (°)	1.53
Ramachandran statistics (Coot)	
Favoured	293 (93.91%)
Allowed	13 (4.17%)
Disallowed	6 (1.92%)

†Rmerge= Σ hΣi │I(h,i)-<I(h)>│/ Σ hΣi I(h,i), where I(h,i) is the intensity of the ith measurement of reflection h and <I(h)> is the mean value of the intensity of reflection h.

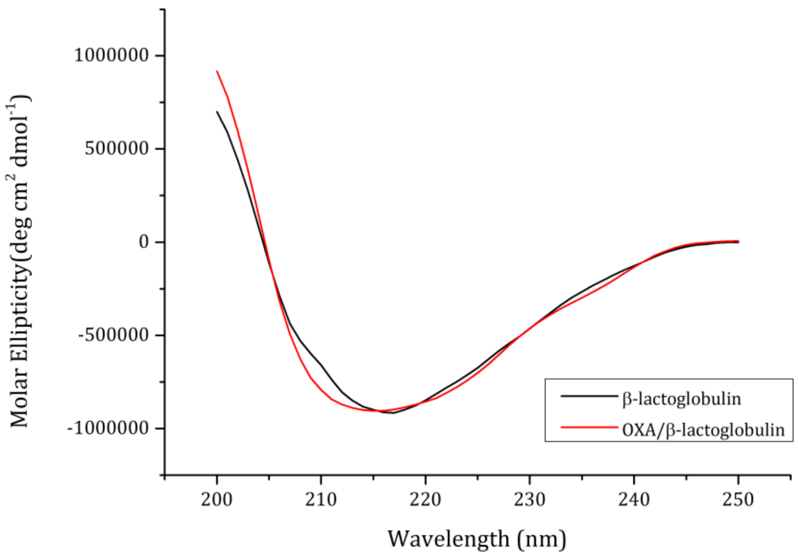

Figure S1. Circular dichroism spectra of OXA/β-lactoglobulin adduct (red line) and of the metal-free protein (black line) in 5mM Hepes pH 7.5. The adduct has been prepared in protein to metal molar ratio 1:5.

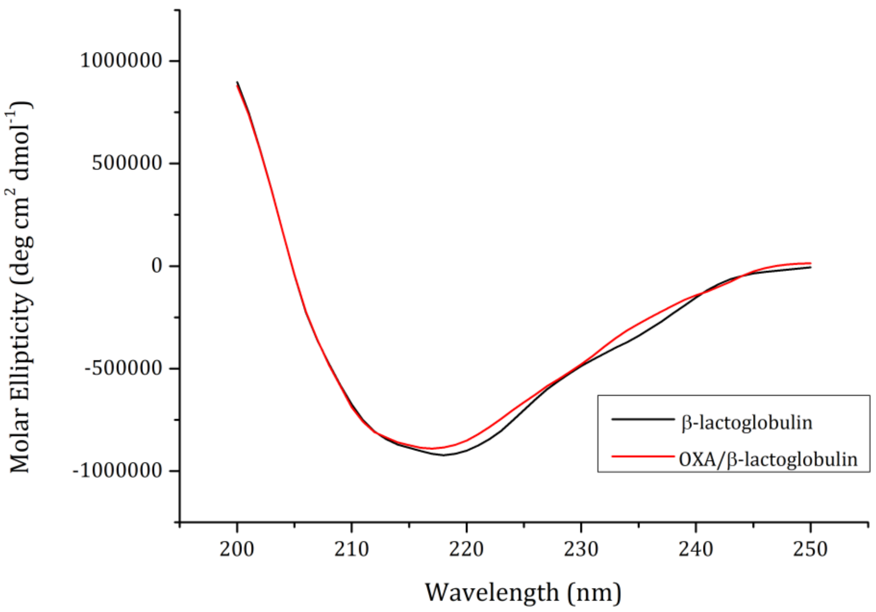

Figure S2. Circular dichroism spectra of OXA/β-lactoglobulin adduct (black) and of metal-free protein (red) in 10 mM ammonium acetate at pH 6.8. The adduct has been prepared in protein to metal molar ratio 1:5.

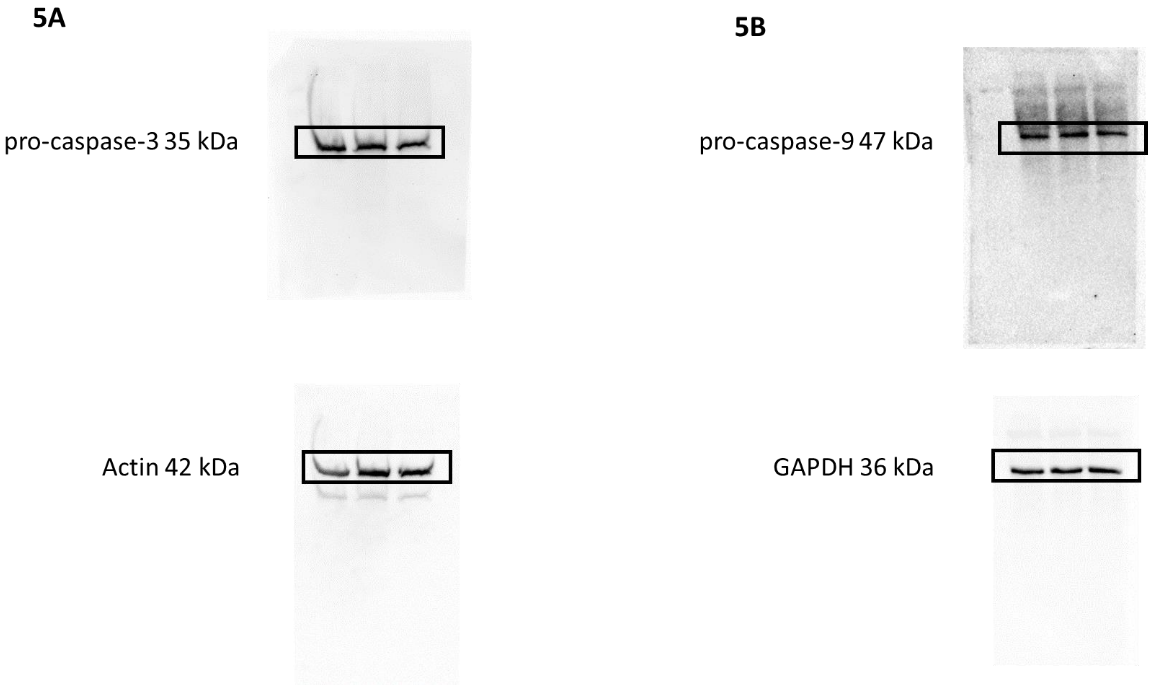

Figure S3. Uncropped image of western blot experiments.

References

1. Essemine, J.; Hasni, I.; Carpentier, R.; Thomas, T.J.; Tajmir-Riahi, H.A. Binding of biogenic and synthetic polyamines to β-lactoglobulin. Int J Biol Macromol 2011, 49, 201–209.
2. Vonrhein, C.; Flensburg, C.; Keller, P.; Sharff, A.; Smart, O.; Paciorek, W.; Womack, T.; Bricogne, G. Data processing and analysis with the autoPROC toolbox. Acta Cryst 2011, D67, 293-302.
3. McCoy, A.J.; Grosse-Kunstleve, R.W.; Adams, P.D.; Winn, M. D.; Storoni, L. C.; Read, R. J. Phaser crystallographic software. J Appl Cryst 2007, 40, 658-674.
4. Balasco, N.; Ferraro, G.; Loreto, D.; Iacobucci, I.; Monti, M.; Merlino, A. Cisplatin binding to β-lactoglobulin: a structural study. Dalton Trans 2020, 49, 12450-12457.
5. Murshudov, N.; Vagin A.A.; Dodson, E.J. Refinement of Macromolecular Structures by the Maximum-Likelihood Method. Acta Cryst 1997, D53, 240-255.
6. Emsley, P.; Lohkamp, B.; Scott, W. G.; Cowtan, K. Features and development of Coot. Acta Cryst 2010, D66, 486-501.
7. Cucciolito, M. E.; D'Amora A.; De Feo G.; Ferraro G.; Giorgio A.; Petruk P.; Monti D. M.; Merlino A.; Ruffo F. Five-coordinate Platinum (II) compounds containing sugar ligands: Synthesis, characterization, cytotoxic activity, and interaction with biological macromolecules. Inorg Chem 2018, 57, 3133-3143.
